# Expression of Antimicrobial Peptide Genes in the Canine Amniotic Membrane

**DOI:** 10.3390/vetsci9050200

**Published:** 2022-04-21

**Authors:** Rajit Lohajaroensub, Chenphop Sawangmake, Channarong Rodkhum, Nalinee Tuntivanich

**Affiliations:** 1 Department of Veterinary Surgery, Faculty of Veterinary Science, Chulalongkorn University, Bangkok 10330, Thailand; rajit.loha@gmail.com; 2 Department of Veterinary Pharmacology, Faculty of Veterinary Science, Chulalongkorn University, Bangkok 10330, Thailand; chenphop.s@chula.ac.th; 3 Department of Veterinary Microbiology, Faculty of Veterinary Science, Chulalongkorn University, Bangkok 10330, Thailand; channarong.r@chula.ac.th

**Keywords:** antimicrobial peptide genes, amniotic membrane, dog, gene expression

## Abstract

The human amniotic membrane has been successfully used in human ocular reconstruction. Several studies have demonstrated its properties, including antimicrobial features. As a result of the restricted availability of human amniotic membrane for veterinary use, canine amniotic membrane has become an attractive alternative. Clinical studies of the application of canine amniotic membrane in animals and the understanding of its biological properties are limited. This study aimed to determine the expression of peptide genes of natural antimicrobials in canine amniotic membrane. Expressions of canine β-defensin 1, 102, and 103, and canine Elafin were determined in healthy puppies by real-time quantitative polymerase chain reaction. Canine β-defensin 1, 103, and Elafin were expressed in all samples, possibly suggesting a role in the innate immune system of normal canine amniotic membrane. Further investigations of protein expression and localization are recommended.

## 1. Introduction

In recent years, the amniotic membrane (AM) has been increasingly used as a biomaterial for tissue reconstruction due to its transparent structure, lack of immunogenicity, and excellent potential as a substrate for wound recovery [1]. In clinical practice, AM has been used in various surgical procedures, such as skin transplantation [2], intrauterine adhesion [3], urinary tract reconstruction [4], pericardial substitution [5], and oral surgery [6]. The use of human AM transplantation in human ophthalmology was first reported to manage in the conjunctival defect in symblepharon patients by De Rötth in 1940 [7]. A study by Kim and Tseng reestablished the use of AM in clinical ophthalmology by carrying out the transplantation of preserved human AM on damaged rabbit corneas for ocular surface reconstruction. Their results suggested a progressive recovery of corneal epithelium and promising vascularization [8]. Since then, there have been several reports on the use of human AM transplants in ocular reconstitution in a variety of ocular pathologies, including chemical and thermal burns [9], bullous keratopathy [10], limbal stem cell deficiency [11], deep corneal ulcers [12], corneal perforation [12], and glaucoma surgeries [9]. In an ophthalmic setting, AM transplantation is typically used to support damaged tissue, protect and shield defects from further degeneration or breakdown due to external factors, reduce pain, and promote healing. 

A number of studies have demonstrated several properties of human AM. The interesting clinical properties include the promotion of wound healing and exhibition of antifibrotic, anti-inflammatory, anti-angiogenic and antimicrobial features [13]. A study using the reverse transcriptase-polymerase chain reaction (RT-PCR) and enzyme linked immunosorbent assay (ELISA) showed that human AM contains several growth factors promoting epithelialization during corneal wound healing [14]. Growth factors include epidermal growth factor, keratinocyte growth factor, hepatocyte growth factor, basic fibroblast growth factor, transforming growth factor-α, and transforming growth factor-β. Not only growth factors, but human AM also expresses anti-inflammatory and antifibrotic mediators, such as cytokines, interleukin-10, inhibin, and activin [15]. Thrombosporidin-1 and tissue inhibitors of metalloproteinases identified by RT-PCR in epithelial and mesenchymal cells of human AM have anti-angiogenic characteristics to reduce vascularization [16]. Human AM contains protease inhibitors which have an inhibitory effect on proteinases in acute corneal alkali burn [17]. 

Human AM also showed antimicrobial activity. In in vitro study, human AM is found to have inhibition properties against various strains of Gram-positive and -negative bacteria, using antimicrobial susceptibility testing methods [18]. Natural antimicrobial peptides are key components of the innate immune system and have broad-spectrum protection against bacteria, viruses, and parasites [18]. Human AM was reported to contain natural antimicrobial peptide genes: human β-defensins (*hBDs*) 1–3 [18,19,20,21,22], Secretory leukocyte proteinase inhibitor (*SLPI*) [18,19], Elafin [18,19,20], and cathelicidin [18,22]. The localization of β-defensins 1–3 and Elafin was found at the amniotic epithelium in human [20]. The BDs are a major family of vertebrate natural antimicrobial peptides, produced mainly by leukocytes and epithelial cells [23]. Whey acidic protein is a group of antimicrobial peptides, which include SLPI and Elafin. Elafin gene expression has been identified in the epithelium of the respiratory tract [24], the intestinal tract [25], and AM [19,20] in humans. Defensins and whey acidic proteins show similar biochemical characteristics and antimicrobial activities, as they interact with microbial membranes via their cationic charge and hydrophobic amino acids, causing the disruption of microbial structure [26,27].

The clinical application of AM in veterinary ophthalmology was first reported by Kim and Tseng [8] in rabbits. It offered progressive and steady epithelialization and sectorial vascularization of the cornea [8]. Clinical applications of human AM have been reported with satisfactory results in horses [28], cats [29], and dogs [30]. The use of human AM graft as a treatment for canine complicated corneal ulcer including of melting cornea and corneal perforation [31] and feline corneal sequestration [29] showed good outcomes. The use of human AM transplantation combined with superficial keratectomy gave much less corneal scarring in equine corneal squamous cell carcinoma [28].

According to the higher demand for AM in veterinary medicine, AM from animals has become attractive due to its availability and accessibility. In 1998, equine AM was the first membrane generated from animals for surgical implantation [32]. The application of equine AM demonstrated rapid healing of various corneal diseases after clinical application to horses, dogs and cats [33,34]. Bovine AM improved canine uncomplicated corneal erosion [35]. Porcine AM transplantation demonstrated effective elimination of tissue debris and regeneration of corneal tissue in canine corneal ulcer [36]. 

Clinical applications of canine AM for therapeutic transplantation were reported in canine bullous keratomalacia [37], canine deep corneal ulcer [38], and large corneal dermoid [39]. Rapid epithelialization without neovascularization and scarring of the cornea were clinically promoted [39]. Research of canine AM as an alternative material that can be potentially used in clinical practice is becoming more popular. It is beneficial that canine AMs are abundance in our research environment as our research group is based at the university’s small animal hospital and veterinary school where the caesarean section is a routine operation. Recently, canine AM has been characterized [40]. The level of epidermal growth factor protein expression found to be maintained in preserved canine AM [41] suggests other biological properties may have been present to some extent. Antimicrobial peptides have the potential to be a viable alternative to standard antibiotics due to their function as endogenous antibiotics. The growth of microbes is disrupted by antimicrobial peptides. This leads to the reduced opportunity for microbes to develop antimicrobial resistance [42]. There is growing interest in the antimicrobial peptide genes that are being studied in canine AM. 

This study aimed to investigate mRNA expression of canine antimicrobial peptides in the AM of healthy dogs using quantitative real-time PCR.

## 2. Materials and Methods

### 2.1. Canine Amniotic Membrane 

Four placentas from healthy puppies were collected after cesarean sections in the operation room, Small Animal Teaching Hospital, Faculty of Veterinary Science, Chulalongkorn University (Bangkok, Thailand). All pregnant dogs (French bulldog, Mixed, and Chihuahua) aged 1–6 years were clinically healthy and had completed a vaccination program. A canine brucellosis test was performed using the Antigen Rapid Canine Brucella Antibody Test Kit (Bionote, Hwaseong-si, Korea). Dogs with a history of abortion or dead fetus, systemic inflammation or infection within 3 months, abnormal blood profile, positive for brucellosis, or signs of inflammation of canine AM were excluded. All puppies in this experiment survived and were confirmed healthy by the owners. Placental tissue and AM in the experiments are in good conditions. There were no lesions on placental tissue or pregnancy complications 

### 2.2. Sample Collection

After the cesarean sections were carried out, the placentas were collected and placed on the prepared sterile tray. They were immediately transported in a collecting box with ice packs to a laminar flow hood. Canine AMs were gently separated from the remaining chorion and rinsed three times with sterile washing solution to remove blood clot and uteroverdin. Sterile washing solution contained sterile 0.01 M phosphate buffered solution (PBS) containing 50 U/mL penicillin and streptomycin, 10 mg/mL neomycin, and 2.5 mg/mL amphotericin B. Rinsing and membrane preparation procedure were performed in a laminar flow hood. In this current study, we performed an exact same collection procedure as mentioned in a previous study [40] and it showed no microbial growth. To avoid the effect of vascularization (if any), the selective non-vascularized part of AM from each dog was cut and stored in 1 mL RNA later™ Solution (Thermo Fisher Scientific, Waltham, MA, USA) at 4 °C overnight, before transferred to −20 °C until extracted. All placentas were kept sterile during the collection and preparation process. All the processes were carried out continuously without any interruption.

### 2.3. RNA Isolation

Each sample was washed with PBS. Then 30 mg of each sample was cut into small pieces and ground with a pestle and mortar in liquid nitrogen to fine powders. Total RNA was extracted using a NucleoSpin^®^ RNA plus kit (Macherey-Nagel, Düren, Germany), according to the manufacturer’s protocols. Isolated RNA was stored at −80 °C until use.

RNA purity and quantity were assessed by spectrophotometric (Titertek-Berthold, Pforzheim, Germany) and fluorometric methods (Qubit; Life Technologies Corp., Carlsbad, CA, USA), respectively. The A260 /A280 ratio reflects RNA purity ranging from 1.8–2.2.

### 2.4. Quantitative Reverse Transcription PCR (RT-qPCR)

Oligonucleotide primers of canine β-defensin (*cBD*) 1, 102, 103, and Elafin (Table 1) with spanned exon-exon junction were designed from the canine genome sequence using Primer Blast software (https://www.ncbi.nlm.nih.gov/tools/primer-blast/ accessed on 19 March 2021). The obtained RNA products were converted to complementary DNA (cDNA) using a reverse transcriptase enzyme kit (ImProm-II™; Promega, Madison, WI, USA). Real-time PCR reaction mixtures contained SYBR™ Green Master Mix (Applied Biosystems™; Thermo Fisher Scientific, Waltham, MA, USA) and specific forward and reverse primers of genes of interest. Glyceraldehyde 3-phosphate dehydrogenase (*GAPDH*) was used as positive internal control. RNase-free water was used as negative control. The qPCR reactions were performed on the CFX Connect Real-Time PCR Detection System (Bio-rad Laboratories, Hercules, CA, USA). The PCR amplifications were carried out as follows: 50 °C for 2 min; 95 °C for 2 min; and 40 cycles of 95 °C for 10 s, 60 °C for 10 s. Amplifications were followed by dissociation (melting) curves to ensure specificity of the primers. The relative mRNA expressions were normalized with the reference gene, *GAPDH*. PCR products were analyzed on 2% TAE agarose gels stained with Novel Juice dye (GeneDireX Inc., Las Vegas City, NV, USA).

### 2.5. Data Analysis

The relative mRNA expressions were normalized with the reference gene according to the following formula: 2^ΔCq^, where ΔCq = [Cq target gene − Cq *GAPDH*]. Cq is referred to the quantification cycle. Data are described and shown as mean ± standard error of the mean (SEM) using SPSS software version 22 (SPSS Inc., Chicago, IL, USA).

## 3. Results

**Figure 1:**
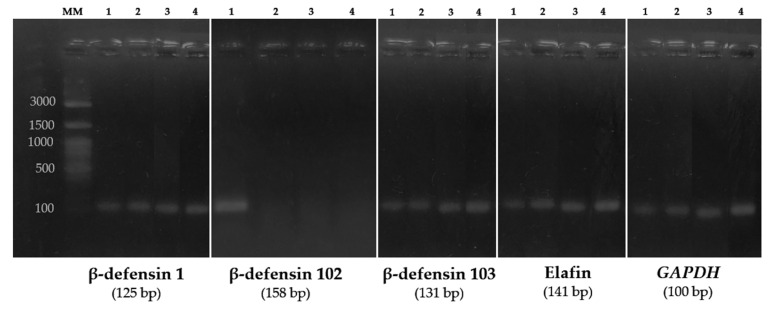
Expression of canine β-defensin and Elafin in canine AM. RT-qPCR products amplified from the cDNA of each dog were fractionated on 2% agarose gels and stained with Novel Juice dye. Numbers represent each canine amniotic membrane sample in each column. MM was molecular size marker (100 bp). *GAPDH* was used as positive internal control (reference gene). RNase-free water was used as negative control. Numbers on the left indicate sizes (bp) of the molecular weight marker.

**Figure 2:**
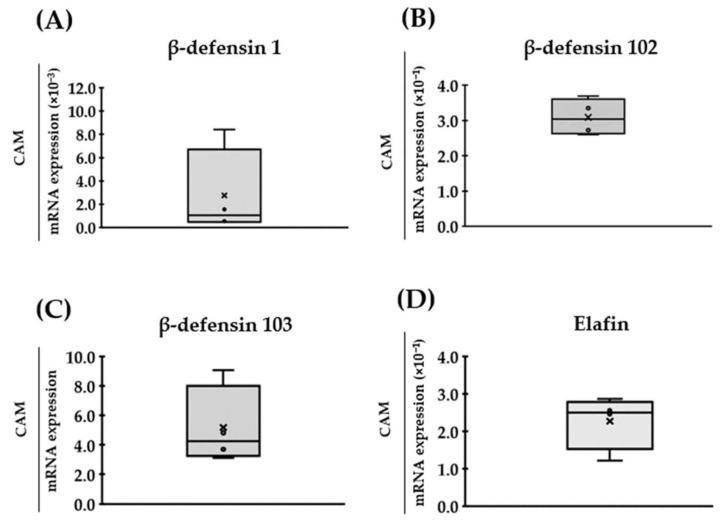
Box plots of Cq value distribution of RT-qPCR; (**A**) β-defensin 1, (**B**) β-defensin 102, (**C**) β-defensin 103, and (**D**) Elafin. Y-axis demonstrates relative mRNA expression between gene of interest and reference gene.

The expressions of *cBD1*, *cBD102*, *cBD103*, and Elafin mRNA were determined in canine AM using RT-qPCR. RT-qPCR amplification demonstrated transcripts for *cBD1*, *cBD103*, and Elafin in all four CAM samples collected from different puppies. Transcripts for *cBD102* were detected in one sample. All genes appeared to have strong and roughly equal transcript levels when compared to *GAPDH* control lanes (Figure 1). The expression of *cBD1* was 2.74 × 10^−3^-fold (±1.91 × 10^−3^) of the reference gene. Expressions of *cBD102*, *cBD103*, and Elafin were 3.09 × 10^−1^ ± 0.26 × 10^−1^, 5.18 × 10^−1^ ± 1.34 × 10^−1^, and 2.27 × 10^−1^ ± 0.36 × 10^−1^, respectively (Figure 2). 

## 4. Discussion

In this study, we demonstrated the gene expressions of *cBD1*, *cBD102*, *cBD103*, and Elafin in canine AM. β-defensins and Elafin genes are major antimicrobial peptide genes showing expression in human amniotic epithelial cells [20]. Recent studies have elucidated the mechanism of action of antimicrobial peptides from the innate immune system and shown that they play important roles in protecting epithelial and mucosal surfaces from microbial attack [26,27]. In addition to antimicrobial actions, both BDs and Elafin serve other functions. Human BDs have chemoattractant activity in the integration of the innate and adaptive immune systems [23], while Elafin inhibits neutrophil elastase and proteinase 3, attenuating several key processes in the inflammatory cascade [27]. 

From the study of Patil [43], *cBD1* and *cBD103* are orthologs with *BD1* and *BD3* reported in humans, by phylogenetic analysis. From sequencing analysis of skin [44], *cBD1* was shown to be a reasonable ortholog to monkey and pig and *cBD103* showed a moderate similarity to mouse, rat, and pig orthologs. Elafin showed modest similarity to bovine, ovine, and primate orthologs. Our gel electrophoresis showed strong mRNA transcriptional levels, which is in accordance with the strong expression of *hBD1–3* and Elafin in primary amniotic epithelial cells, reported by Stock [19]. Localization of hBD1–3 and Elafin was revealed by immunohistochemistry in amnion epithelium [20], while hBD3 was found in fibroblasts in amnion mesenchyme. The investigation of cBD1–3 and Elafin localization in AM is suggested for further study.

Although *BD* family gene expression is found in various organ systems in vertebrates, the expression patterns differ among species. In avion, chicken *BD* genes (*Gal1*–*Gal13*) are identified. Seven of them are found expressed in bone marrow and epithelial cells of respiratory tract. Another six genes are expressed specifically in the liver and reproductive tract. Reproductive-specific *BD* genes are expressed in the testis and vas deferens in males and in the ovaries and oviduct in females [45].

In rats, *BD* genes are grouped into four clusters. Most of them are expressed predominantly in the rat testis and epididymis. Some other *BD* genes, such as *BD1* and *BD42*, are expressed in the spleen and kidney. *BD24* is specifically expressed in the ovary. *BD36* is expressed in the inner surface of mucosal respiratory, gastrointestinal, and reproductive tracts [43].

In pigs, *BD* mRNA expression are found abundantly in tissues that required extent mucosal defenses. Porcine tissues found *BD* mRNA expression predominantly and diversely include kidney, testis, respiratory tract, small intestine epithelium, spleen, and thymus. However, some *BDs* seem to be specific to developing tissue, such as *pBD115*, which is expressed in infant testis but not in adults. This suggests a specific role of the *BD* in the developmental biology of distinct tissue or physiological systems [46].

In cow, *BD* genes are categorized into four clusters (Cluster A, B, C, and D) as well as in rats and pigs. Cluster A consists of four *BD* genes and is highly conserved among mammals. However, the roles and expression of *BD* in this cluster have not yet been studied in cows. *BD* genes in cluster B are expressed predominantly in the reproductive tract. Cluster C is the cluster that arises from cluster A, the function and expression pattern of which have not been yet studied in cows. Cluster D is the largest family of *BD* in cows and also the most ancient cluster among mammals. The expressions of *BD* genes in this cluster seem to be specific to the immune system, respiratory system, and epithelial cells of various tissue [47].

In canines, *cBD103* expression was found via RT-qPCR in skin, tongue, lung, epididymis, testes [48] and the nasal cavity [49]. We report here the significantly highest expression of *cBD103*, followed by *cBD102*, Elafin, and *cBD1,* respectively. Our result is comparable to the study of Buhimschi [21], which found that *hBD3* expression was significantly more abundant compared to *hBD1* and *hBD2* in human amniotic epithelial cells. They also showed that *hBD3* was upregulated in response to the microbial mimic lipopolysaccharide and peptidoglycan, while *hBD1* and *hBD2* levels were unchanged. Potent *hBD3* mRNA expression after this treatment demonstrated significant bactericidal activity over other β-defensins against Gram-positive and -negative bacteria. In our study, the strong expression of canine Elafin in canine AM appeared to be important as reported in normal human AM [18,19,20], as well as normal canine skin and testes [44]. Protein levels of human Elafin were significantly increased by IL-1β treatment, confirming the secretory state of amniotic antibacterial ingredients [22]. 

King [20] showed the presence of Elafin in human amnion epithelium by immunohistochemistry staining. According to the fact that Elafin is a whey acidic protein, inhibiting serine peptidase, known as a virulent factor of *P. aeruginosa*, further investigation to localize the presence of canine Elafin and canine BDs is recommended. Among various mRNA expression levels of *hBD1–3* and Elafin in primary culture amnion epithelial cells, expression becomes significantly upregulated on treatment with IL-1β [19]. Human BD2 protein level showed dramatically high regulation. Therefore, it will also be interesting to examine antimicrobial peptide expression in canine AM with inflamed or infected conditions. 

The expression of *cBD1* was the least among β-defensin peptides investigated in this study. In various normal canine tissues, *cBD1* expression was detected in the skin [44], trachea [50], and testes [51]. However, the expression level of *cBD1* in testis and lung are 400-fold and 100-fold higher than the expression in small intestine, respectively [51]. In a culture of canine tracheal epithelial cells, low expression of *cBD1* mRNA levels was found, but this was not statistically significantly increased after lipopolysaccharide treatment [50]. Likewise, the production of *hBD1* mRNA was generally important because there was no change of gene expression in human AM after treatment by IL-1β [20]. This suggests that *cBD1* plays little or no role in the innate defense mechanism of normal AM. Although previous studies suggest mild antimicrobial activities of cBD1, studies on synthesized cBD103 have revealed promising antimicrobial properties in culture environments [49,50]. This may explain the much higher detection of *cBD103* than *cBD1* in this research, and that cBD103 is essential in CAM to inhibit bacterial growth in the tissue.

Canine *BD102* is not the canine ortholog of *hBD2,* but it is instead specific to the dog [43]. Among antimicrobial peptides investigated in our study, *cBD102* mRNA was not uniformly expressed in all samples. Inconsistent expression of *cBD102* mRNA was also observed in normal canine skin [44] and canine lymph node [52]. A different level of gene expression was also found in canine normal and atopic dermatitis at various sites [53]. A study of Sang [51] and Leonard [48] showed strong expression of *cBD102* in the testes compared to no expression in bone marrow, intestine, liver, kidney, spleen, and lung.

In a comparative study on the expression of *cBD1*, *cBD102*, *cBD103*, *cBD122* and *cBD124* in skin, variations were observed in normal dogs among 11 different dog breeds [53]. The variation in gene expression may possibly be influenced by various factors, such as breeds [53], age [54], maternal dietary and parity [55], and the defensin copy number in individual animal. In aging Beagles, a comparative study on immunity detected a significant decrease of the bacterial killing ability of neutrophils in older dogs. In addition, the mRNA expression level of some immune genes was decreased due to aging factors [54]. Though we cannot conclude that breed and age could influence the quality of antimicrobial peptide in our study, it will be interesting for a further study to investigate the effects of breed and age on antimicrobial ability and immune system. 

The study of these genes here is essential to propose the potential of canine AM as an alternative material for human AM in clinical use as a wound healing graft. One of the limitations of this study is that we studied only three out of 43 β-defensins in canines [43] and only Elafin from the whey acidic group [56]. Nevertheless, there are still other antimicrobial peptides that may play a role in canine AM. For example, *SLPI* and cathelicidin are also well-known antimicrobial peptides and interesting candidates for future study. It also must be noted that gene expressions do not always directly reflect protein abundance. Some mechanisms alter the protein quantity after transcription and translation. For example, post-transcriptional modification of mRNA plays an essential role in this. The mechanism splices mRNA and reconstructs it before the translation. Moreover, post-translational modification is another mechanism that could alter the function of proteins after the syntheses [57]. Thus, although the gene expressions of proteins of interest are observed, their antimicrobial activities may not be relevant to the expression. The regulation of *BD* mRNA expression in canine AM by the cellular response to inflammation and infection remains unknown. To detect antimicrobial peptide expression and concentration in canine AM, the use of ELISA or Western blot may provide greater insight regarding protein abundance and quantity. Moreover, immunoprecipitation is another potential tool to investigate the activity of protein of interest. This method could detect the interaction between proteins and help us to understand their roles in some antimicrobial pathways. In addition, a minimum inhibitory concentration (MIC) test would contribute to our understanding of the required dosage of antimicrobial proteins to inhibit the growth of bacteria. Taken together, the study of the gene expression of antimicrobial peptides in canine AM would shed light on the potential application of this tissue in medical use as a grafting material for would healing and the prevention of bacterial infection.

## 5. Conclusions

We have demonstrated the presence of *cBDs* and canine Elafin expression in the canine AM of healthy dogs. Canine β-defensins 1, 103, and Elafin may play an important role in the innate immune system of normal canine AM.

## Figures and Tables

**Table 1:** Primer sequences for RT-qPCR.

Gene	Accession Number	Sequences	Primer Pairs	Tm (°C)	Length (bp)
*cBD1*	NM_001113713	Forward	ATGAGGCCTCTCTACTTGCTG	59.24	125
		Reverse	CCTCCTTTCCTGGCACAGATG	60.68	
*cBD102*	NM_001113715	Forward	CCCTGAGTTTGTCAACCATGA	58.14	158
		Reverse	CCGGTTATGAGGGCATCTGAAT	60.22	
*cBD103*	NM_001129980	Forward	GCCTGTTGGTCATGAGGATCT	59.79	131
		Reverse	GCACCGACCGCTCCTTATT	60.15	
Elafin	NM_001290099	Forward	TTCTTGGTCCTGGCAGTGTT	59.45	141
		Reverse	CGGATCTCGACCTCTAACCG	59.41	
*GAPDH*	NM_001003142	Forward	CCAACTGCTTGGCTCCTCTA	59.38	100
		Reverse	GTCTTCTGGGTGGCAGTGAT	59.67	

*cBD*, canine β-defensin; *GAPDH*, Glyceraldehyde-3-phosphate dehydrogenase.

## Data Availability

The data are available upon request from the submitting author.
